# Two fabricated carbon paste electrodes for novel potentiometric determination of probenecid in dosage form and human plasma

**DOI:** 10.1038/s41598-022-24920-0

**Published:** 2022-11-28

**Authors:** Mahmoud A. Tantawy, Israa A. Wahba, Samah S. Saad, Nesrin K. Ramadan

**Affiliations:** 1 Analytical Chemistry Department, Faculty of Pharmacy, Cairo University, Kasr El Aini Street, Cairo, 11562 Egypt; 2 Chemistry Department, Faculty of Pharmacy, October 6 University, 6 October City, Giza, Egypt; 3 Pharmaceutical Analytical Chemistry Department, College of Pharmaceutical Sciences and Drug Manufacturing, Misr University for Science & Technology, 6Th of October City, Giza, Egypt

**Keywords:** Electrochemistry, Analytical chemistry

## Abstract

Solid contact ion selective electrodes are extensively utilized owing to their marvelous performance over traditional liquid contact ones. The main drawback of those solid contact electrodes is aqueous layer formation which affects their constancy. Herein and to overcome this common drawback, a carbon paste electrode containing poly(3,4-ethylenedioxythiophene) was constructed and used for determination of probenecid at variant pH values. This modification decreased the potential drift down to 0.8 mV/h and improved its stability over 30 days. A Nernstian slope of − 57.8 mV/decade associated with a linear range of 1.0 × 10^−6^–1.0 × 10^−2^ mol/L was obtained. The modified carbon paste electrode successfully detected up to 8.0 × 10^−7^ mol/L probenecid. Results of this modified carbon paste electrode were also compared to unmodified one.

## Introduction

Potentiometric technique is widely used for the electrochemical determination of organic ions in an aqueous solution without the need for prior separation step^1,2^. In this technique, two types of electrodes are commonly employed, namely; liquid contact ion selective electrode and solid contact one (SC-ISE). The latter is usually easily handled and fabricated (e.g. using a carbon paste), economically feasible, and widely applicable compared to liquid contact one^3–8^. However, SC-ISEs suffer from main obstacle related to the creation of an aqueous layer between the lower solid support and the upper ion selective polymeric matrix. This layer is associated with potential irreproducibility and irregular potential drift, as it serves as a tank for electrolytes and thus disturbing sample composition^9^.

To overwhelm this drawback, a hydrophobic conductive layer is usually incorporated at a solid contact/polymeric ion sensitive membrane interface. Conducting polymers, such as; poly(3,4- ethylenedioxythiophene) (PEDT), polyaniline, polypyrrole, and poly(benzopyrene), are generally utilized to limit the aqueous layer formation. They also have a redox active capacitance and a polymeric elastic nature^10–12^. The highly conjugated and porous PEDT possesses the ability to reduce electrochemical impedance as well as to enhance charge injection significantly^7^. It also exhibits stability at a wide working pH range and in the existence of carbon dioxide or oxygen^13–15^. These features candidate PEDT for various applications, such as; conductive coatings, electroluminescence, electrolytic capacitors, photovoltaics^13^, bioelectronics^16^, and potentiometric analyses of inorganic ions^14,15^ and different drugs in many matrices^7,17,18^.

**Figure 1:**
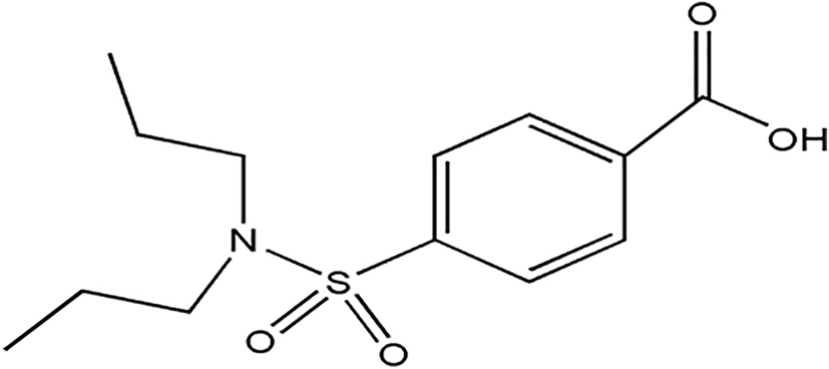
Chemical structure of probenecid.

Probenecide (PRO) is a derivatized sulfonamide drug with a chemical designation of 4-(dipropyl sulfamoyl) benzoic acid^19^, Fig. 1. It enhances uric acid excretion and is hence utilized to treat gout as well as gouty arthritis. PRO inhibits urate reabsorption at kidney proximal tubules facilitating its excretion and lowering serum urate concentration^20^. Regarding the former decade, some methods have been reported for the analysis of PRO in its bulk powder or dosage forms. Those comprise various chromatographic methods^21–23^ and spectrophotometric ones^24,25^. To the best of our knowledge, none of the reported methods have described its potentiometric determination. Therefore, we aimed to design two carbon paste electrodes (CPEs) for the first potentiometric determination of PRO. A PEDT modified and unmodified CPEs were developed and compared. The modified electrode was applied for quantification of the studied drug in pure powder, combined tablets, and spiked human plasma.

## Results and discussion

### Performance characteristics of PEDT-modified and unmodified CPEs

For developing ISEs, many factors are considered, for instance, the nature of the drug, ion exchanger lipophilic characters, and the used additives, like plasticizer^26,27^. pKa value of PRO≈3.5, therefore, it behaves as an anion at pH more than 4.5. Hence, TDAB was chosen as an anion exchanger to be incorporated into ion sensitive membrane cocktail^28^. It is noted that maximum selectivity and sensitivity were attained upon using TDAB in amount equivalent to 5.0 mg. Possible membrane saturation might be responsible for no additional enhancement upon adding larger amounts^1^. A PRO soaking solution of 1.0 × 10^−4^ mol/L was utilized in order to precondition the prepared membrane. Br^−^ ion substitution with the anionic PRO occurred during this phase^28^. Plasticizer also plays an important role to make the membrane malleable as well as improving components distribution. Furthermore, it helps the membrane permittivity allowing the anionic drug to transfer easily between the aqueous sample phase and hydrophobic membrane one^29^. In this work, NPOE was the chosen plasticizer due to its extraordinary lipophilic characteristics.

**Figure 2:**
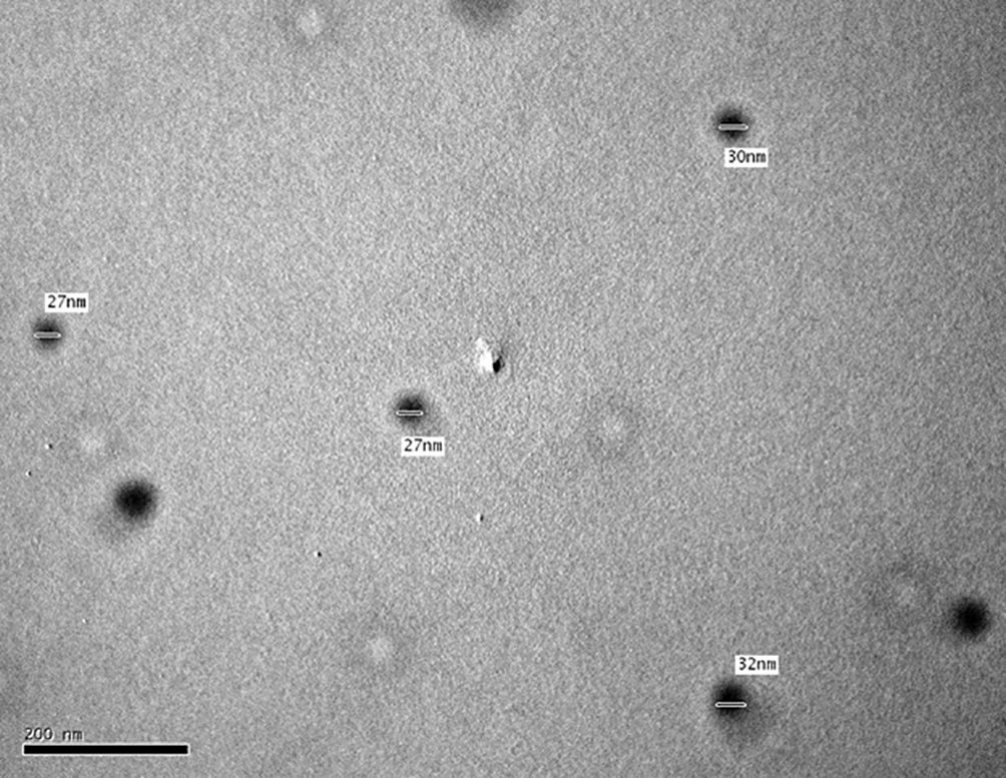
TEM imaging of PEDT dispersion.

**Figure 3:**
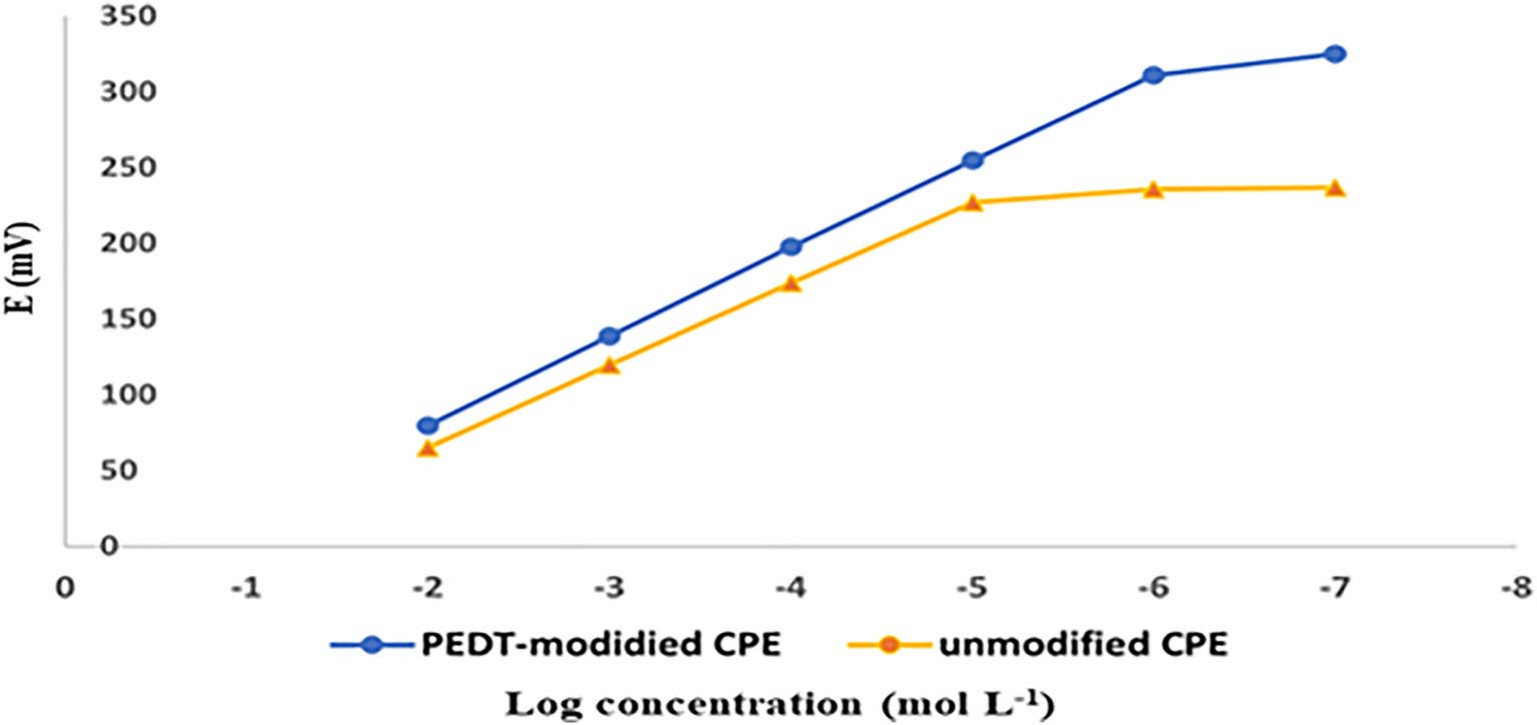
Potential profile (in millivolts) to the logarithm of concentrations of probenecid (1.0 × 10^−7^–1.0 × 10^−2^ mol/L) using PEDT-modified CPE and unmodified CPE.

The target of this work is to develop two CPEs and to estimate the impact of PEDT on modified sensor constancy. Firstly, the size, shape, and distribution of utilized PEDT nanoparticles were examined via TEM imaging to guarantee good electrical conductivity associated with their large surface area. An average diameter of 30 nm with good nanoparticles distribution was watched, Fig. 2. After that, the performance characteristics of PEDT-modified CPE versus unmodified CPE were assessed according to the IUPAC^30,31^, Table 1. Two calibration plots were constructed with respective linearity ranges of 1.0 × 10^−6^ to 1.0 × 10^−2^ and 1.0 × 10^−5^ to 1.0 × 10^−2^ mol/L, Fig. 3. The acquired slopes for PEDT-modified and unmodified CPEs were − 57.8 and − 54.0 mV/decade, respectively. The enhanced sensitivity of PEDT-modified CPE may be attributed to its ion-to-electron transduction ability and hydrophobicity leading to the prevention of anionic drug ions from being trapped in the inner aqueous layer^7,18^.

**Table 1:** Electrochemical response characteristics of the proposed PRO electrodes.

Parameter	PEDT-modified CPE	Unmodified CPE
Slope (mV/decade)	− 57.8	− 54.0
intercept (mV)	− 34.6	− 42.5
LOD (mol/L)^a^	8.0 × 10^−7^	9.0 × 10^−6^
Response time (s)	5	10
Working pH	5.0–9.0	5.0–9.0
Range (mol/L)	1.0 × 10^−6^–1.0 × 10^−2^	1.0 × 10^−5^–1.0 × 10^−2^
Correlation coefficient (r)	0.9999	0.9999
Lifetime (days)	30	23
Accuracy^b^	99.76 ± 0.739	–
Repeatability^c^	1.394	–
Intermediate precision^c^	1.790	–
Reproducibility^c^	3.023	–
Robustness^d^	1.574	–

^a^Detection limit was estimated by interception of the extrapolated arms of calibration curves. ^b^Mean ± SD of recoveries for five determinations. ^c^RSD% of recoveries for the determination of three concentrations (1.0 × 10^−5^, 1.0 × 10^−4^ and 1.0 × 10^−3^ mol/L), repeated three times within the day for repeatability, repeated in three successive days for intermediate precision and measured using three different electrodes for reproducibility (*n* = 9). ^d^RSD% of recoveries for the determination of two concentrations (1.0 × 10^−4^ and 1.0 × 10^−3^ mol/L) measured at three different buffer pH values of 6.5, 7.0 and 7.5 (*n* = 6).

Both sensors were investigated through evaluating their long-term potential stability. PEDT-modified CPE showed a drift in the potential of ≈0.8 mV/h whereas it significantly increased to ≈7.0 mV/h for the unmodified one, Figure S1, supplemental information. The observed high potential/time drift for the unmodified electrode is due to water layer formation^7^. Additionally, PEDT-modified CPE’s slope did not exhibit any notable changes during thirty days of consecutive usage (SD ≈2.2) which ensures the positive influence of PEDT on electrode sensitivity. A rapid response time of about five seconds was also observed upon recording the required time for each concentration to reach a stable potential reading of ± 1 mV, Figure S2, supplemental information. This may be attributed to the established ionic equilibrium at PEDT-modified CPE/aqueous solution interface^18^. In nutshell, the modification of CPE with PEDT enhances the constancy and sensitivity of such electrode as opposed to an unmodified one.

### Aqueous layer test

**Figure 4:**
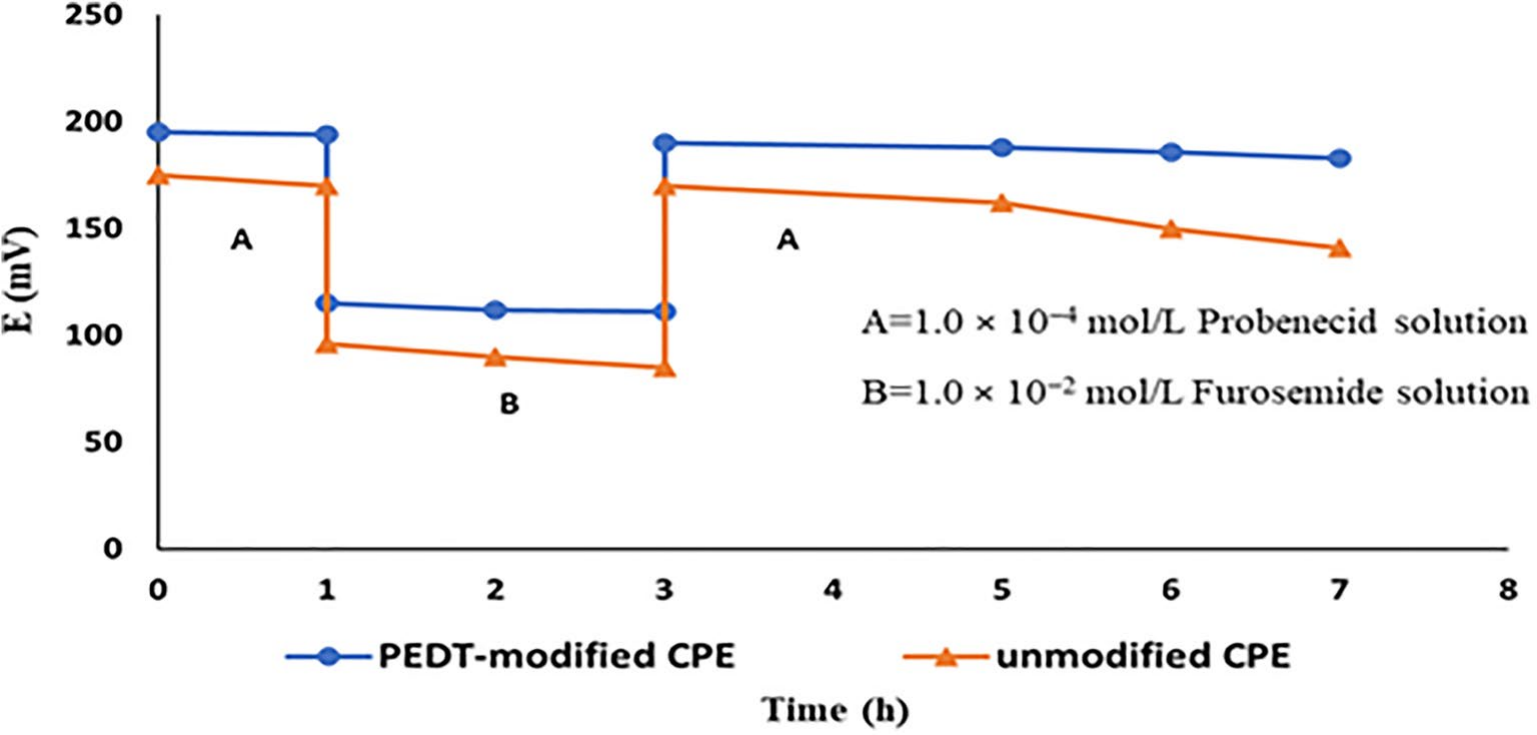
Water layer test for PEDT-modified CPE and unmodified CPE where potential in (mV) was recorded in (A) 1.0 × 10^−4^ mol/L probenecid solution and (B) 1.0 × 10^−2^ mol/L furosemide solution.

This test depends on detecting water layer formation. Potential measurements were first carried out in 1.0 × 10^−4^ mol/L PRO then subjecting the two tested CPEs to structurally close interfering ion of higher concentration (1.0 × 10^−2^ mol/L). The two electrodes were finally returned again to 1.0 × 10^−4^ mol/L PRO. In this test, furosemide was the chosen interfering drug. PEDT-modified CPE exhibited an almost null potential drift besides adequate reversibility. Notable drifts were alternatively witnessed for the unmodified one, Fig. 4. The potential drift of unmodified CPE is attributed to the replacement of PRO by furosemide leading to alteration in the composition of ions through membrane ions flow^28^.

### Soaking time effect

Five calibration plots were constructed at different time intervals starting from 1 to 24 h. The soaking time of 24 h was the most appropriate at which a − 57.8 mV/decade slope was obtained. On the other hand, a time of more than 24 h is not desirable for electrode soaking as electroactive species may leach^28^.

### pH study

**Figure 5:**
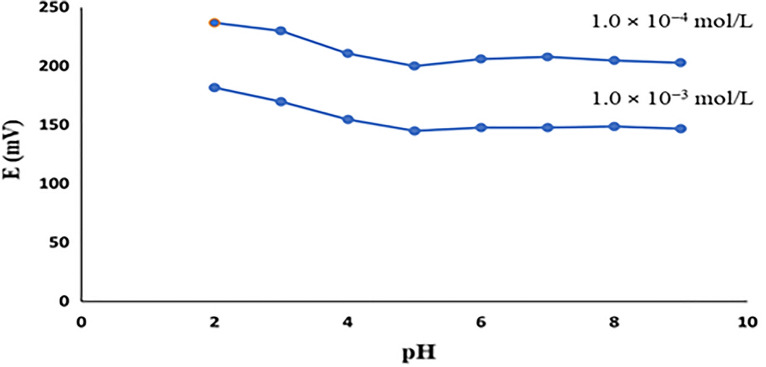
pH effect on response of the PEDT-modified CPE.

To study the pH effect, the performance of the proposed PEDT-modified CPE was examined at different pH values (2.0 to 9.0) to obtain the optimum experimental conditions. Potentials for 1.0 × 10^−3^ and 1.0 × 10^−4^ mol/L PRO were measured through using different Britton Robinson buffers as solvents, Fig. 5. The sensor response was fairly constant at pH ≥ 5.0 proving the ionization of PRO with the existence of drug negative form (carboxylate ion). Gradual increase in the potential was rather observed at a pH lower than 5.0. This phenomenon may be attributed to molecular/ionized PRO ratio alteration^28^.

### Temperature effect

For temperature effect on the proposed PEDT-modified CPE, a range of 25–35 °C was selected whereas readings of 1.0 × 10^−4^ mol/L PRO were recorded, Figure S3, supplemental information. The observed potential readings suggested a reasonable thermal constancy up to 35 °C as a quite stable potential was obtained without affecting the slope and response time upon increasing the temperature.

### Selectivity studies

Selectivity of the prepared PEDT-modified CPE in presence of structurally correlated furosemide, inorganic ions, namely; Cl^−^, Br^−^, carbonate, phosphate, and sulphate, and some common inactive ingredients, was checked by separate solution method (SSM)^32^. The same concentrations of 1.0 × 10^−4^ mol/L, for analyte and interferents, were utilized in this study. The obtained results of the estimated selectivity coefficients revealed that the electrode is highly selective to PRO with no noteworthy interference from possible interferents, Table 2.

**Table 2:** Logarithmic selectivity coefficients (K ^pot^_PRO, interferent_) of PEDT-modified CPE.

Interferent	log (K ^pot^_PRO, interferent_)^a^
Furosemide	− 1.16
Cl^−^	− 2.78
Br^−^	− 2.53
Carbonate	− 2.67
Phosphate	− 2.86
Sulphate	− 2.82
Glucose	− 3.31
Starch	− 3.27
Sodium glycolate	− 2.88
Magnesium stearate	− 2.94
Microcrystalline cellulose	− 3.34

^a^Average of three determinations.

### Combined tablets and human plasma applications

PRO concentration was successfully estimated in Goutyless® tablets using the suggested PEDT-modified CPE. It is worth noting that the co-formulated colchicine as well as the added excipients did not interfere with the intact drug assay. The calculated mean recovery was found to be 99.27% ± 1.621 (*n* = 3). For human plasma application, acceptable results have been acquired for the examination of PRO-spiked samples (98.18% ± 3.825, *n* = 9). As noticed, the proposed PEDT-modified CPE owns good capability for successful determination of PRO in combined Goutyless® tablets as well as biological samples with no need for additional procedures to remove co-formulated colchicine, additives, ions, or plasma proteins effects.

### Statistical tests

The mean and variance of the proposed method were compared with those of official titrimetric one^33^ via student’s t-test and F-test, respectively, Table S1, supplemental information. No significant difference, concerning accuracy and precision, was detected as the calculated values were lower than the theoretical ones at a confidence interval within 95%. Moreover, the proposed CPE is simpler to use and needs less equipment compared to the official titration method.

## Conclusion

The proposed PEDT-modified CPE overcame the common drawback of the potential instability of SC-ISEs associated with water layer formation. Its electrochemical performance characteristics were compared with the unmodified one. The modified sensor exhibited remarkably shorter response times and improvement in potential stability. Results of the potentiometric aqueous layer test indicated that the hydrophobicity of PEDT guarded against water layer formed between the polymeric membrane and the carbon paste support. The investigated analytical method had superiority of being low cost, sensitive, fast, time saving, and selective for probenecid determination compared to the official one. The method was effectively applied for the potentiometric quantitative determination of probenecid in a pharmaceutical formulation as well as human plasma.

## Methods

### Materials and reagents

A pure sample of PRO was obtained from October Pharma, Egypt. Potency was checked using the British Pharmacopoeia (BP) assay method^33^ and was found to be 100.72% ± 1.073. Goutyless® tablets (500 mg PRO & 0.5 mg colchicine per tablet, batch no. B05210619; October Pharma, Egypt) were purchased from the local market. Tetradodecylammonium bromide (TDAB, ≥ 99.0% (AT)), PEDT dispersion in water, 2-nitrophenyl octyl ether (NPOE, ≥ 99.0%), tetrahydrofuran (THF, ≥ 99.9%), polyvinyl chloride (PVC), graphite powder (≤ 20 µm) and multi-walled carbon nanotubes (MWCNTs) with ≥ 98% carbon basis, 10 ± 1 nm O.D, 4.5 ± 0.5 nm I.D & 36 µm L were obtained from Sigma-Aldrich, Germany. Blank human plasma samples were purchased from VACSERA, Egypt. Britton Robinson buffer was prepared via mixing equal volumes of acetic, boric and phosphoric acids (0.04 mol/L each) where pH was adjusted using 0.2 mol/L sodium hydroxide.

### Instruments

pH and potential measurements were performed through a Jenway pH meter (3510; Essex, UK) using Metrohm pH glass electrode (No. 6.0133.100; Herisau, Switzerland) and Thermo Fisher Scientific double-junction silver/silver chloride reference electrode (Orion 90–02; MA, USA), respectively. Teflon-body CPE working electrode (MF-2010, BN: 003,051,907, BASi; IN, USA), with a 2.87-mm cavity, 1-mm deep, and a copper wire for electrical connection, was employed. Characterization of PEDT particle size was accomplished using transmission electron microscopy (TEM) technique; JEOL (JEM, 1230; Tokyo, Japan).

### Electrodes fabrication

The employed carbon paste was obtained through mixing 8.0 g graphite and 2.0 g MWCNTs powders with 4.3 g paraffin oil in a mortar for about 30 min^8^. CPE cavity was packed with this paste. For the PEDT-modified electrode, 10.0 µL PEDT dispersion was drop-casted on the electrode surface and left to dry at ambient temperature for 10 min. 10.0 µL of membrane cocktail dispersion was then drop-casted for deposition of polymeric ion sensing membrane. This dispersion was formed through the addition of 200.0 mg PVC, 0.4 mL NPOE and 5.0 mg TDAB to 5.0 mL THF followed by sonication for 10 min. The investigated electrodes were preconditioned through soaking in 1.0 × 10^−4^ mol/L PRO for 24 h at a pH of 7.0.

### Potentiometric measurement

1.0 × 10^−2^ mol/L stock solution was prepared by weighing 71.34 mg probenecid. This amount was then dissolved in the least amount of Britton Robinson buffer pH 7.0. The solution was finally transferred to a 25-mL volumetric flask, and the volume was completed to mark with the same buffer. Serial dilutions were performed to prepare working solutions with a concentration range of 1.0 × 10^−7^ to 1.0 × 10^−2^ mol/L. Those dilutions were prepared through transferring 2.5 mL from each preceding concentration into sets of 25-mL volumetric flasks, then completing volumes with buffer pH 7.0. The adopted CPEs were conjoined with a double junction silver/silver chloride reference electrode, and potentiometric measurements were conducted. Potential difference values were noted, and two calibration curves were plotted.

### Application to Goutyless® tablets

Five Goutyless® tablets were powdered and an amount equivalent to 50.0 mg PRO was weighed. The powder was cautiously introduced to a 25-mL volumetric flask, and shaken for 10 min with about 10 mL Britton Robinson buffer pH 7.0. Buffer was added to complete the volume to the mark. 2.5 mL was then delivered into a new 25-mL flask, and diluted to the mark using the same buffer. A final suspension of a concentration claimed to be 7.0 × 10^−4^ mol/L was prepared. The proposed PEDT-modified CPE was then dipped in that solution along with the reference one. Potential difference reading was documented, and the respective concentration was calculated.

### Application to human plasma

2.5 mLs of three PRO standard solutions (1.0 × 10^−2^, 1.0 × 10^−3^ & 1.0 × 10^−4^ mol/L) were independently transferred into three 25-mL flasks. Volumes were then diluted to the mark using plasma. Potential difference readings were directly recorded with no pretreatment procedures taking into consideration that a dilution factor of 10 was implied.

## Supplementary Information

Supplementary Information.

## Data Availability

The data that support the findings of this study are available from the corresponding author upon reasonable request.
