# Supplementary material for: Two fabricated carbon paste electrodes for novel potentiometric determination of probenecid in dosage form and human plasma
Source: Sci Rep. 2022 Nov 28;12:20418. doi: 10.1038/s41598-022-24920-0 (PMC9705367; doi:10.1038/s41598-022-24920-0)
Supplement: Supplementary file 1 — Supplementary Information. [file 41598_2022_24920_MOESM1_ESM.docx]

Supplemental Information

For

**Two fabricated carbon paste electrodes for novel potentiometric determination of probenecid in dosage form and human plasma**

Mahmoud A. Tantawy, Israa A. Wahba, Samah S. Saad, Nesrin K. Ramadan


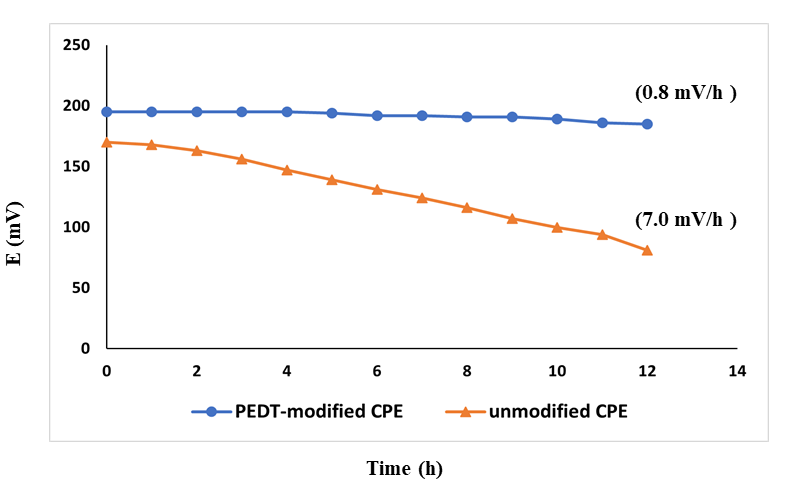


Figure S1. Potential stability of the proposed sensors over 12 h for a 1.0 × 10^−4^ mol/L solution of probenecid.


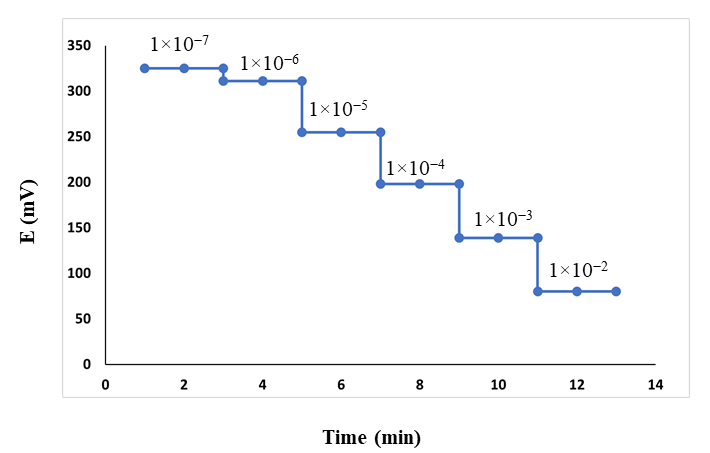


Figure S2. Potential as a function of time in dynamic response plot**.**


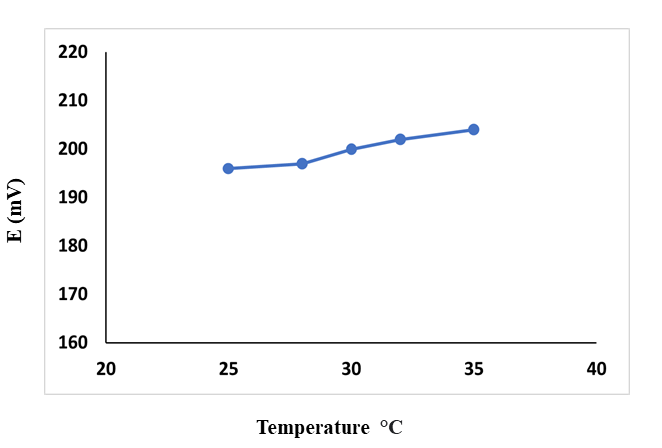


Figure S3. Effect of Temperature on PEDT-modified CPE (1.0 × 10^−4^ mol/ L).

Table S1. Statistical comparison of obtained results from proposed and official methods.

| **Value** | **PEDT-modified CPE** | **Official method^a^** |
| --- | --- | --- |
| **Mean** | 99.76 | 100.72 |
| **SD** | 0.737 | 1.073 |
| **n** | 5 | 5 |
| **V (variance)** | 0.543 | 1.151 |
| **Student's-t test ( 2.306) ^b^** | 1.994 | - |
| **F-test (6.388) ^b^** | 2.119 | - |

^a^ Official titrimetric method: ethanol was used as a solvent then titrating against 0.1 M sodium hydroxide, endpoint was determined potentiometrically.

^b^ The values in parenthesis are the corresponding theoretical values of t and F at p = 0.05.
